# *FANCA* Gene Mutations in North African Fanconi Anemia Patients

**DOI:** 10.3389/fgene.2021.610050

**Published:** 2021-02-19

**Authors:** Abir Ben Haj Ali, Olfa Messaoud, Sahar Elouej, Faten Talmoudi, Wiem Ayed, Fethi Mellouli, Monia Ouederni, Sondes Hadiji, Annachiara De Sandre-Giovannoli, Valérie Delague, Nicolas Lévy, Massimo Bogliolo, Jordi Surrallés, Sonia Abdelhak, Ahlem Amouri

**Affiliations:** ^1^Department of Histology and Cytogenetics, Institut Pasteur de Tunis, Université Tunis El Manar, Tunis, Tunisia; ^2^Laboratory of Biomedical Genomics and Oncogenetics, Institut Pasteur de Tunis, Université Tunis El Manar, Tunis, Tunisia; ^3^INSERM, MMG, UMR 1251, Aix Marseille University, Marseille, France; ^4^Department of Peadiatric Immuno-Haematology, National Bone Marrow Transplantation, Tunis, Tunisia; ^5^Haematology Department, Hedi Chaker Hospital, University of Sfax, Sfax, Tunisia; ^6^Research Institute IIB Sant Pau, Hospital de la Santa Creu i Sant Pau, Universitat Autonoma de Barcelona, Barcelona, Spain; ^7^Centro de Investigación Biomédica en Red de Enfermedades Raras (CIBERER), Instituto de Salud Carlos III (ISCIII), Madrid, Spain

**Keywords:** consanguinity, founder mutations, North Africa, molecular diagnosis, Fanconi anemia, *FANCA*

## Abstract

Populations in North Africa (NA) are characterized by a high rate of consanguinity. Consequently, the proportion of founder mutations might be higher than expected and could be a major cause for the high prevalence of recessive genetic disorders like Fanconi anemia (FA). We report clinical, cytogenetic, and molecular characterization of *FANCA* in 29 North African FA patients from Tunisia, Libya, and Algeria. Cytogenetic tests revealed high rates of spontaneous chromosome breakages for all patients except two of them. *FANCA* molecular analysis was performed using three different molecular approaches which allowed us to identify causal mutations as homozygous or compound heterozygous forms. It included a nonsense mutation (c.2749C > T; p.Arg917Ter), one reported missense mutation (c.1304G > A; p.Arg435His), a novel missense variant (c.1258G > A; p.Asp409Glu), and the *FANCA* most common reported mutation (c.3788_3790delTCT; p.Phe1263del). Furthermore, three founder mutations were identified in 86.7% of the 22 Tunisian patients: (1) a deletion of exon 15, in 36.4% patients (8/22); (2), a deletion of exons 4 and 5 in 23% (5/22) and (3) an intronic mutation c.2222 + 166G > A, in 27.3% (6/22). Despite the relatively small number of patients studied, our results depict the mutational landscape of FA among NA populations and it should be taken into consideration for appropriate genetic counseling.

## Introduction

Fanconi Anemia (FA) is a rare, inherited disorder clinically characterized by various congenital abnormalities and a predisposition to develop malignancies, especially acute myeloid leukemia, and other cancers (Kutler et al., 2003). The prognosis of the disease is characterized by an evolution toward progressive bone marrow failure (BMF). Without treatment, FA is most often lethal before the end of the second decade of life. Cells from FA patients present high levels of spontaneous chromosomal breakages (Schroeder and Kurth, 1971) that increase dramatically by clastogens such as Mitomycin C (MMC; Cervenka et al., 1981) and Diepoxybutane (DEB; Auerbach, 2009).

FA is genetically heterogeneous, with at least 22 genes encoding for a group of proteins that cooperate in a unique FA/BRCA DNA repair pathway (Nalepa and Clapp, 2018). Indeed, the products of these genes are involved in the preservation of genome integrity in response to physiological stress or to genotoxic agents (Kottemann and Smogorzewska, 2013; Ceccaldi et al., 2016). All FA genes are located on autosomal chromosomes except *FANCB* which is X-linked (Kitao and Takata, 2011). *FANCA*, *FANCC*, and *FANCG* complementation groups include, respectively, 60, 15, and 10% of all FA patients from the USA, whereas mutations in other FA genes occur less frequently (<1–4%; Neveling et al., 2009).

Several reports indicated a higher FA prevalence in some ethnic groups like the Spanish Romani (Callén et al., 2005; Castella et al., 2011), the Afrikaner population of South Africa (Tipping et al., 2001), Ashkenazi Jewish (Whitney et al., 1993), and Tunisian populations (Amouri et al., 2014) due to founder effects and isolation. Thus, information on a patient’s ethnic origin may provide evidence for a pathogenic mutation that is likely to be causal (Faivre et al., 2000; Tipping et al., 2001; Kutler and Auerbach, 2004; Castella et al., 2011). In Tunisia, FA-A complementation group is the most prevalent, with a 94% frequency among the FA patients (Bouchlaka et al., 2003). A common haplotype shared by FA patients mostly originating from the South of Tunisia was associated with the deletion of exon 15 in *FANCA* (Bouchlaka et al., 2003; Amouri et al., 2014). Another founder mutation in the same gene (c.890_893del in exon 10) was identified in Tunisian Jewish FA patients (Tamary et al., 2000). In neighboring countries, Algeria and Libya, no FA molecular studies have been conducted so far. However, several studies on other genetic conditions have shown that patients originating from North Africa (NA) shared many common founder mutations (Richard et al., 2008; Messaoud et al., 2010; Bensenouci et al., 2016). According to more than 20 years’ experience on the investigation of genetic diseases, identification of founder mutations that are shared between Tunisia and other NA countries even with a single patient were of great impact for launching molecular diagnosis in their respective countries. This was the case for *Xeroderma pigmentosum* (Senhaji et al., 2013) and for several other diseases (Romdhane et al., 2012; Charoute et al., 2015).

The present study aims to investigate the *FANCA* mutational spectrum and to develop a strategy based on molecular approaches applicable to routine clinical use for FA patients originating from NA.

## Materials and Methods

A total of 87 families with 113 FA patients have been addressed to our department in Institut Pasteur de Tunis (IPT) for confirmation of FA based on the standard test involving MMC-induced chromosomal breakage analysis. The cytogenetic and molecular status of 30 families with 46 patients have been previously described (Talmoudi et al., 2013; Amouri et al., 2014). Since FA is a rare disease, it was difficult to recruit a large number of patients. Under these circumstances, the conduct, the analysis and the interpretation of studies on this disease may sometimes be limited to different degrees by its prevalence. Also, we have to take into consideration the severe health status of the patients in particular in regard to their extremely severe anemia, especially that we give the priority to diagnosis (cytogenetic study), thus most of the patients were investigated cytogenetically but no sufficient material was available to conduct molecular investigation. Furthermore, most of the cases are pediatric cases and we try to avoid any invasive tests including recurrent blood sampling. Therefore, we were not able to collect additional samples for DNA investigation. Finally, transplanted and recently transfused patients were excluded from the study. As a direct outcome of these, the present study was conducted on 22 newly collected families, including 29 FA patients who agreed to participate in the molecular genetic testing.

This study was conducted according to the principles of the Declaration of Helsinki and was approved by the biomedical ethics committee (2017/14/I/LR11IPT05/V0) of Pasteur Institute. Informed consent of the legal patients’ representatives were obtained. Peripheral blood samples were collected from the 29 FA patients, their parents, and their siblings when available.

Genomic DNA was extracted using the standard salting-out method (Miller et al., 1988) or the QIAamp DNA mini kit (Qiagen).

Clinical examination and familial information details were recorded. Clinical data were obtained based on history, physical examinations, and reviewing of medical records.

Patients were initially screened using PCR to check the homozygous deletion of the exon 15 in the *FANCA* gene (LRG_495) which is a common founder mutation in Southern Tunisia (Amouri et al., 2014).

The 43 coding exons of *FANCA* gene were amplified using PCR. Intronic oligonucleotides primer pairs were generated using Primer 3^1^ and in silico PCR (UCSC).^2^ Direct sequencing of PCR products was performed on an ABI prism 3,500 DNA Genetic Analyzer (Applied Biosystems, Foster City, CA, USA), using the ABI Prism Big Dye Terminator v3.1 Cycle Sequencing Ready Reaction Kit (Applied Biosystems). Sequences were then analyzed using Bioedit software and compared to the wild sequence using BLAST program available in the NCBI server.

Multiplex ligation-dependent probe amplification (MLPA) was performed to detect large deletions or duplications within the *FANCA* gene using a SALSA P031-A2/P032 kit (MRC-Holland BV, Amsterdam, The Netherlands) for (F068/15, TFA27, SFA15, F076/15, F031/15, F032/15, and F050/15) patients. PCR products were analyzed using ABI PRISM 3130 Genetic analyzer (Applied Biosystems).

For (TFA27, SFA15, F059/15, F058/15, CFA4, CFA3, F033/15, and TFA23) patients, available family members were genotyped with three microsatellites flanking the *FANCA* gene (D16S3026‐ D16S3121‐ D16S3407) as previously reported (Bouchlaka et al., 2003).

Targeted gene sequencing (TGS) has been conducted for (F072/09, SFA2, GF06/09, and GF78/09) patients. Custom design of the genes’ panel was performed using SureDesign (Agilent Technologies Inc.); probes were generated to cover the exons and 15 bp of the surrounding intronic sequences of a total of 87 candidate genes known to be involved in DNA repair disorders. Library preparation for NGS was performed using Agilent’s HaloPlex^HS^ (high sensitivity) workflow as a target enrichment method. Amplicon libraries were prepared from patients’ genomic DNA using the HaloPlex^HS^ PCR target enrichment system dedicated to Ion Torrent PGM according to the manufacturer’s recommendations. Massively parallel sequencing was performed on an Ion Torrent PGM (Thermo Fisher Scientific). In our analysis we used the Ion Torrent Suite Software v.4 to process our data and we configured the settings to reduce the total number of false positive indels caused by the high frequency of homopolymer sequencing errors (min_cov_each_strand: 0, min_variant_score: 10, min_allele_freq: 0.1, snp_min_coverage: 6 snp and indel; strand_bias: 0.98 snp and 0.85 indel). In addition, we visually evaluated the read depth and the percentage of mutated reads for each variant using the Integrative Genomic Viewer (IGV). Finally, validation by MLPA was made to confirm the identified indels. Data were analyzed using the in-house VarAft software version 2.5, which is available online.^3^ We prioritized rare functional variants (missense, nonsense, splice site variants, and indels) and excluded variants with a Minor Allele Frequency (MAF) > 0.01 in dbSNP137 and 138, in the Exome Variant Server,^4^ 1000 Genomes Project,^5^ or Exome Aggregation Consortium database (ExAC), Cambridge, MA.^6^ A number of online tools were used to predict the functional impact and pathogenicity of the variants such as MutationTaster,^7^ PolyPhen,^8^ SIFT,^9^ I-mutant,^10^ and CADD.^11^

## Results

In this study, we report on the clinical, cytogenetic, and molecular investigation of 22 families including 29 FA patients. Our cohort is composed of 8 girls and 21 boys (sex ratio = 2.625) aged from 1 to 28 years. Inbreeding examination showed that 25 patients (86.20%) were born from consanguineous unions. Eight families are multiplex (having more than one affected child). Geographical distribution showed that five patients are from Libya, one from Algeria, one Algerian/Tunisian patient (father: from Algeria; mother: from Tunisia) and 22 are Tunisians. The latest group (Tunisian patients) is composed of 14 patients originating from Southern Tunisia, six from Central Tunisia and two from Northern Tunisia.

Most of patients presented typical clinical features of FA including: skin pigmentation abnormalities (86%), microcephaly (52%), triangular face (31%), and skeletal malformations (34%). Other clinical abnormalities, such as ear abnormalities and hearing problems were presented in two patients (F032/16 and F076/15).

Chromosomal breakage analysis was successful in all patients except for two patients (GF78/09 and SFA2), for whom blood cell culture showed no growth. For these two patients, results of the molecular analysis confirmed the FA diagnosis. For the others, cytogenetic results revealed a high frequency of chromosomal breakages (>3 breaks/cell in MMC treated, up to 0.2 breaks/cell in untreated) in 27 patients when compared to controls (0.1 break/cell in MMC treated, up to 0.03 breaks/cell in untreated).

The molecular approach allowed us to identify seven different pathogenic variants at homozygous and heterozygous states. These variants include large deletions of one or two exons (deletion of exon 15, deletion of exons 4 and 5) respectively, one nonsense mutation (c.2749C > T; p.Arg917Ter) in exon 28, a small deletion (c.3788_3790del; p.Phe1263del) in exon 38, one likely pathogenic missense variant (c.1304G > A; p.Arg435His) in exon 14 that was already associated to FA (Moghrabi et al., 2009), a previously reported intronic mutation c.2222 + 166G > A (Bouchlaka et al., 2003) in intron 24, and a novel missense variant of uncertain significance (c.1258G > A; p.Asp409Glu) in exon 14. For variant c.1258G > A; p.Asp409Glu, no functional study, nor cDNA analysis has been performed, however, the reported minor allele frequency (MAF) in GnomAD exomes (0.00000796) and GnomAD Genomes (0.0000319) and in silico evaluations with predictive algorithms (SIFT, POLYPHEN2, Align GVGD) indicate its possible pathogenicity.

**Figure 1:**
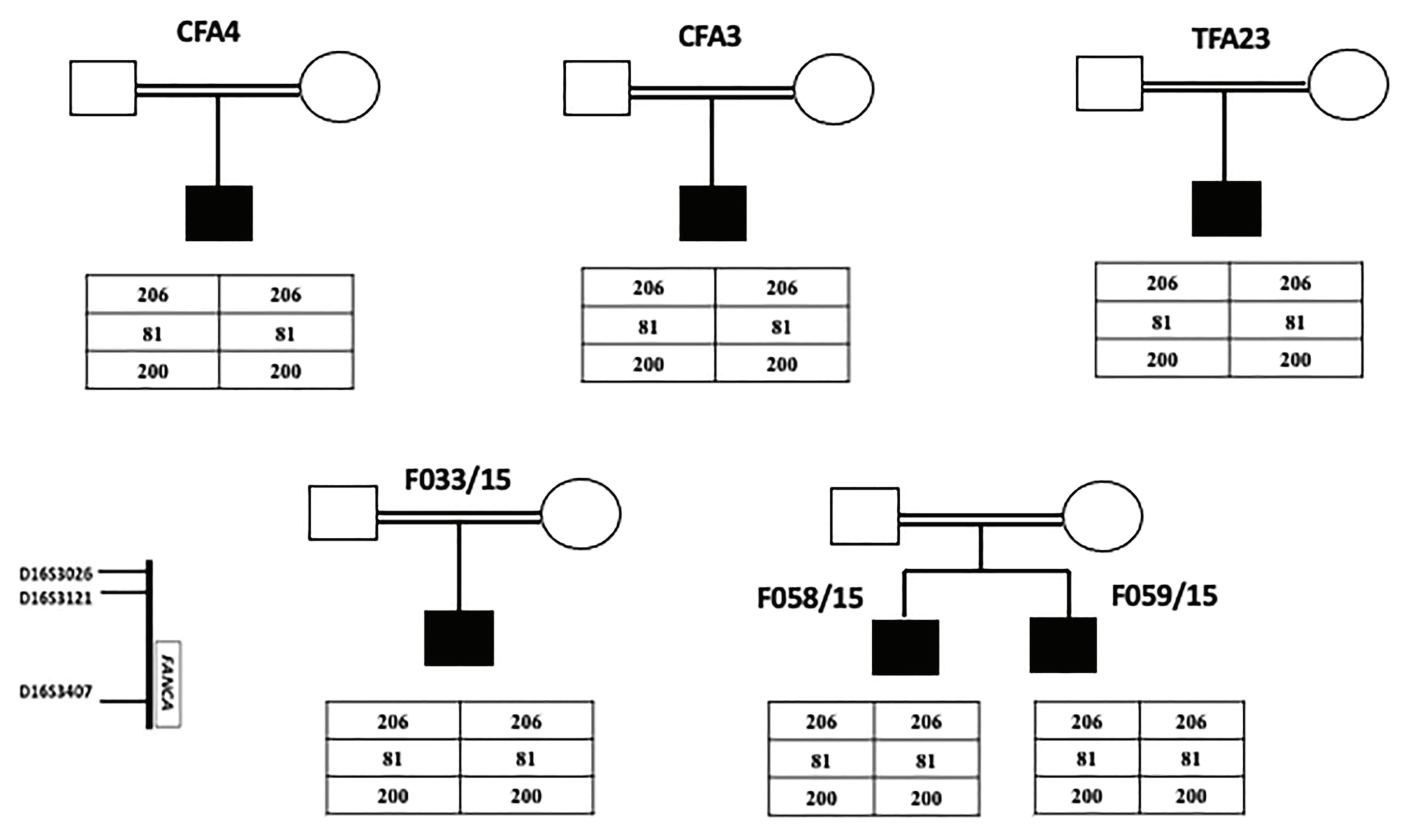
Pedigrees of FA nuclear families originating from Tunisian center (Kairouan) showing genotyping results of three microsatellites markers flanking *FANCA* gene. The presence of a common haplotype in all patients indicates a founder effect.

The last intronic mutation was inherited at a homozygous state in all FA patients originating from Kairouan (Central Tunisia). Furthermore, all these patients shared the same haplotype (206-81-200; Figure 1).

All patients from Sidi Bouzid and Gafsa were homozygous for the founder mutation, deletion of exon 15, except for one patient originating from Gafsa (GF14/12) and another Libyan patient (F075/03) who were both homozygous for the novel missense variant c.1258G > A; p.Asp409Glu.

**Figure 2:**
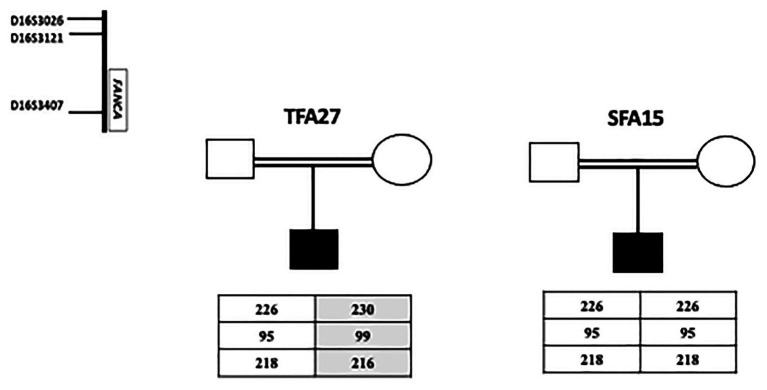
Haplotype analysis for a Libyan SFA15 and an Algerian TFA27 patients. The Libyan patient SFA15 carries homozygously the founder haplotype (226-95-218) already described among Tunisian patients as associated with exon 15 deletion. The Algerian patient TFA27 carries both the founder haplotype associated with exon 15 deletion in the heterozygous state and another haplotype (230-99-216).

Haplotype findings showed that the patient (SFA15) shares the same homozygous haplotype described to be associated with the founder deletion of exon 15 in Tunisian patients (Amouri et al., 2014). On the other hand, the same haplotype was identified at a heterozygous state in the Algerian patient (TFA27) carrying the deletion in one allele. This patient is probably compound heterozygous. These results suggest a common ancestor between Tunisian, Libyan, and Algerian FA patients bearing the exon 15 deletion but do not exclude the hypothesis of another ancestor, especially for Algerians (Figure 2).

Deletion of exons 4 and 5 has been identified in five patients originating from Sfax. Four of them belong to two related families (family 10 and family 11) and were homozygous for this large deletion. The other patient (GF06/09) was heterozygous and the second mutation is still unknown.

**Figure 3:**
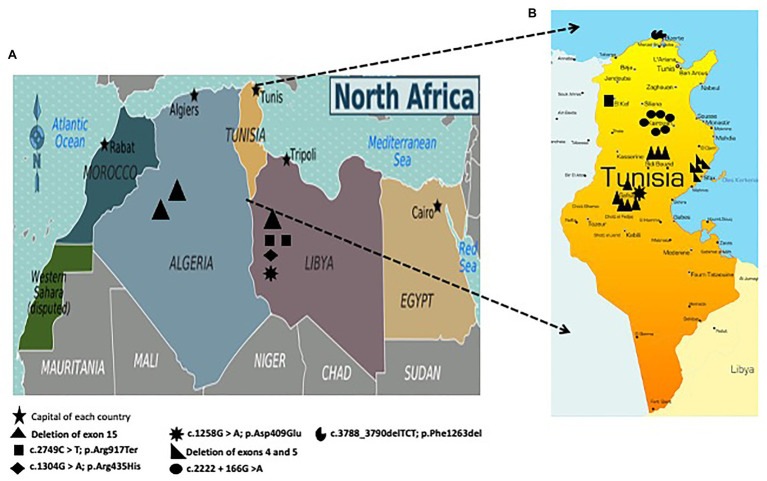
**(A)** Geographic distribution of mutations in the *FANCA* gene among Algerian and Libyan patients; **(B)** Geographic distribution of mutations in the *FANCA* gene including three founder mutations among Tunisian patients: c.2222 + 166G > A in Kairouan (Central Tunisia), deletion of exon 15 in Sidi Bouzid (Southern Tunisia) and Gafsa (Southern Tunisia), and deletion of exons 4 and 5 in Sfax (Southern Tunisia).

Our findings showed that deletion of exon 15, the large deletion of exons 4 and 5, and the intronic mutation c.2222 + 166G > A are founder mutations in Sidi Bouzid/Gafsa, Sfax, and Kairouan, respectively (Figure 3).

A summary of genealogical, clinical, cytogenetic, and molecular characteristics of the 29 studied patients is presented in Table 1.

**Table 1:** Clinical, cytogenetic, and genetic features in North African Fanconi anemia patients.

Family number	Patient code	Sex/Age (years)	Parents relatedness	Origin	Clinical manifestations	Percentage of chromosomal instability (CI %)	Type of mutations	Zygosity	Detection methods
Family 1	F068/15	F/10	No	Algeria/Tunisia (Kef)	-Hyperpigmentation ‐Microcephaly ‐Thrombocytopenia ‐Anomalies of the thumb	100%	(c.2749C > T; p.Arg917Ter) Deletion of exon 15	CH	MLPA/DS
Family 2	TFA27	M/1	2^nd^ degree	Algeria	‐Microcephaly -Hyperpigmentation	70%	Deletion of exon 15	Hz	MLPA/DS
Family 3	F031/16	F/5	No	Libya	‐Thrombocytopenia ‐Café au lait spots	100%	(c.2749C > T; p.Arg917Ter)	Hz	MLPA/DS
	F032/16	M/6			-Ear abnormalities -An extra thumb -Café au lait spots	100%	(c.2749C > T; p.Arg917Ter)	Hz	MLPA/DS
Family 4	F075/03	M/8	1^st^ degree	Libya	-Microcephaly -Hyperpigmentation	90%	(c.1258G > A; p.(Asp409Glu))	Hm	DS
Family 5	GF78/09	M/2	1^st^ degree	Libya	-Thrombocytopenia -Ecchymotic tasks ‐A triangular facies -Red hair -Hypoplasia of the right thumb	No growth of blood cell culture	(c.1304G > A; p.Arg435His)	Hm	TGS
Family 6	SFA15	M/11	2^nd^ degree	Libya	‐Microcephaly ‐Thrombocytopenia ‐Anomalies of the thumb	100%	Deletion of exon 15	Hm	MLPA/DS
Family 7	GF09/09	F/28	1^st^ degree	North of Tunisia (Mateur)	‐A triangular facies -Hyperpigmentation	98%	(c.3788_3790delTCT; p.Phe1263del).	Hm	DS
	GF27/09	F/17			‐A triangular facies ‐Café au lait spots	100%			
Family 8	GF14/12	M/5	No	South of Tunisia (Gafsa)	‐Microcephaly ‐Café au lait spots	100%	(c.1258G > A; p.(Asp409Glu))	Hm	DS
Family 9	GF06/09	M/19	1^st^ degree	South of Tunisia (Sfax)	-Facial dysmorphism -Café au lait spots	90%	Deletion of exons 4 and 5	Hz	TGS
Family 10	F072/09	F/8	1^st^ degree	South of Tunisia (Sfax)	‐Facial dysmorphism -Red hair ‐Hyperpigmentation	100%	Deletions of exons 4 and 5	Hm	TGS
	SFA2	F/7			-Red hair -Facial dysmorphism ‐Anomalies of the thumb -Café au lait spots -Hyperpigmentation -Short stature	No growth of blood cell culture			
Family 11	F076/15	M/19	1^st^ degree	South of Tunisia (Sfax)	-Café au lait spots -Hyperpigmentation -Microcephaly -Triangular facies ‐Microphthalmia ‐Microtia ear	83.6%	Deletions of exons 4 and 5	Hm	MLPA/DS
	F050/15	F/6			-Toe abnormalities -Triangular facies -Red hair	100%			
Family 12	F073/15	M/10	1^st^ degree	South of Tunisia (Gafsa)	-Triangular facies -Café au lait spots	100%	Deletion of exon 15	Hm	DS
Family 13	F022/17	M/10	1^st^ degree	South of Tunisia (Sidi Bouzid)	-Microcephaly ‐Hyperpigmentation	100%	Deletion of exon 15	Hm	DS
Family 14	F008/17	M/12	1^st^ degree	South of Tunisia (Gafsa)	-Café au lait spots -Pre-axial polydactyly	100%	Deletion of exon 15	Hm	DS
Family 15	F492–1/2016	M/11	1^st^ degree	South of Tunisia (Gafsa)	-Microcephaly -Triangular facies ‐ Hyperpigmentation	100%	Deletion of exon 15	Hm	DS
	F492–2/2016	M/9			-Microcephaly -Triangular facies -Café au lait spots	100%			
Family 16	F025/17	F/9	1^st^ degree	South of Tunisia (Sidi Bouzid)	-Triangular facies -Café au lait spots -Anomalies of the thumb	90%	Deletion of exon 15	Hm	DS
Family 17	F059/16	M/7	1^st^ degree	South of Tunisia (Sidi Bouzid)	-Microcephaly -Thrombocytopenia -Anomalies of the thumb	81%	Deletion of exon 15	Hm	DS
	F060/16	M/5			-Café au lait spots -Microcephaly	90%			
Family 18	F058/15	M/9	1^st^ degree	Center of Tunisia (Kairouan)	-Microcephaly -Café au lait spots -Anomalies of the thumb	85%	c.2222 + 166G > A	Hm	DS
	F059/15	M/5			-Microcephaly -Café au lait spots ‐Anomalies of the thumb	92%			
Family 19	CFA4	M/6	1^st^ degree	Center of Tunisia (Kairouan)	-Microcephaly -Café au lait spots	100%	c.2222 + 166G > A	Hm	DS
Family 20	CFA3	M/4	1^st^ degree	Center of Tunisia (Kairouan)	-Triangular facies -Café au lait spots	100%	c.2222 + 166G > A	Hm	DS
Family 21	F033/15	M/3	1^st^ degree	Center of Tunisia (Kairouan)	-Café au lait spots	100%	c.2222 + 166G > A	Hm	DS
Family 22	TFA23	M/8	1^st^ degree	Center of Tunisia (Kairouan)	-Microcephaly -Café au lait spots	100%	c.2222 + 166G > A	Hm	DS

CH, compound heterozygous; Hm, homozygous; Hz, heterozygous; M, male; F, female; DS, direct sequencing; MLPA, multiplex ligation-dependent probe amplification; TGS: targeted gene sequencing.

References for described mutations:
Solanki et al., 2016: c.2749C > T; p.Arg917Ter Amouri et al., 2014: Deletion of exon 15 Moghrabi et al., 2009: c.1304G > A; p.Arg435His Magdalena et al., 2005: c.3788_3790delTCT Ameziane et al., 2008: Deletion of exons 4 and 5 Bouchlaka et al., 2003: c.2222 + 166G > A

## Discussion

This study has been carried out with 29 FA patients. Their clinical features vary from one patient to another, but the common characteristics were: short stature, microcephaly, skin pigmentation, and skeletal abnormalities. Clinically, we found that facial dysmorphia was a common feature in the majority of our patients. This could be related to the predominance of the *FANCA* gene. Indeed, a correlation study between the phenotype of FA patients and the different complementation groups revealed a significantly higher frequency of facial dysmorphia in patients from groups A, G, and C (Faivre et al., 2000). Similarly, congenital malformations (skeletal and skin) were observed in most patients. This is not surprising since a previous study conducted on patients with two founder mutations (deletion of exon 15 and the intronic mutation) showed that malformations were present in 96% of cases and consisted mainly of skeletal malformations. Skin pigmentation abnormalities were present in 92% of cases (Bouchlaka et al., 2003). In addition, an epidemio-clinical study carried out on Tunisian FA patients showed that skin, head, and skeletal abnormalities were common among these patients (90, 71, and 51%), respectively (Hadiji Mseddi et al., 2012).

Clinical heterogeneity within the same family or between patients having the same mutation was also observed in our study. This could be explained by environmental, genetic, and/or epigenetic factors (Koc et al., 1999). It should be noted that there is little genotype–phenotype correlation, apart from the greater severity of group D2 and the association of groups D1/BRCA2 and N with the occurrence of multiple and very early cancers (Mialou, 2011).

In addition, a few previous publications have examined the correlations between the types of specific *FANCA* mutations and phenotype. In fact, a Spanish study showed that no association was found between the type of *FANCA* mutations and hematological diseases or somatic malformations (Castella et al., 2011). The discrepancies observed between these studies may reflect specific characteristics of the population, making it difficult to rely on the Fanconi group or the type of mutation to define the risk of disease complications.

Our results at the clinical and cytogenetic level showed the absence of any correlation between the severity of the disease and the level of cellular sensitivity to MMC which fits with the observations reported in the literature (Castella et al., 2011; Zen et al., 2011; Talmoudi et al., 2013). Therefore, the cytogenetic approach can be considered as an important diagnostic tool for aplastic anemia, especially for asymptomatic cases for whom absence of clinical signs and any familial history would made the clinical diagnosis very challenging, hence showing the importance of chromosomal breakage assessment for the establishment of an accurate and rapid diagnosis. Indeed, testing for induced chromosomal breakage is commonly used for a precise diagnosis in the frame of a proper management of FA patients (bone marrow transplant) and their families (genetic counseling and prenatal diagnosis). Though molecular methods are developed to screen FA patients, cytogenetic investigations with MMC and DEB induction is still “the gold standard” for diagnosing FA.

As showed with previous reports, *FANCA* is the most frequent gene (94%) associated with FA in Tunisia (Bouchlaka et al., 2003). This result suggests that *FANCA* could be also the most causal gene of FA in other NA countries like Algeria and Libya considering their common genetic and historical background.

As previously shown, the deletion of exon 15 in the *FANCA* gene is a founder mutation present at a frequency of 54% among FA-A Tunisian patients (Amouri et al., 2014). This deletion was found to be the most frequent mutation in our cohort (11/29 patients: 37.93%). All the Tunisian patients bearing this deletion were from Sidi Bouzid and Gafsa. This is not surprising as we have previously reported that the geographic distribution of 85% of families bearing this deletion are from Southern Tunisia thus suggesting a founder effect (Amouri et al., 2014). Furthermore, FA cases of Maghrebian origin living in France (Amouri et al., 2014) and Spain (unpublished data) shared this deletion. Our findings, together with what we published in 2014, represent strong evidence that this deletion is the most common mutation among NA population and this observation has to be taken into consideration mainly for migrated families. Similar findings have been observed in NA countries for several diseases. For instance, congenital myasthenic syndrome patients living in France and originating from Tunisia, Algeria, Morocco, and Libya share the same haplotype associated with the point founder mutation (c.1293insG) in *CHRNE* which has been also found in patients from the Maghreb (Richard et al., 2008).

**Figure 4:**
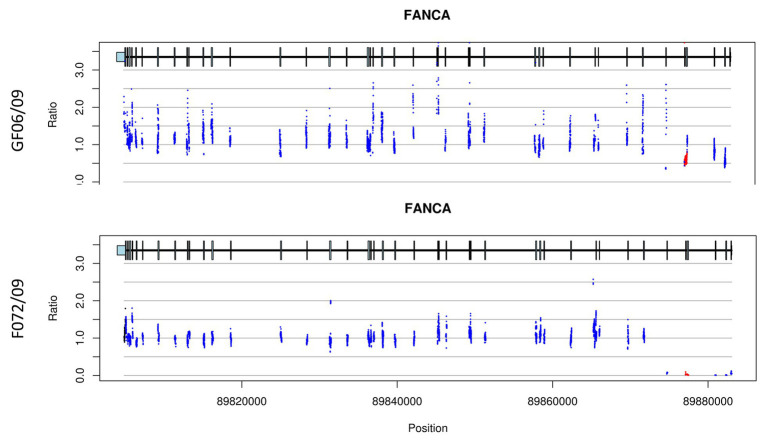
CNV plots for patients (GF06/09 and F072/09) showing exon 4 and 5 deletion. The plots indicate a heterozygous deletion for patient GF06/09 while it is homozygous for patient F072/09. The two plots also show a decrease of the exon 1 signal but the quality control does not retain it as a deletion.

Another large deletion including exon 4 and 5 has been found in patients originating from Sfax (17%; Figure 4). This deletion was firstly described in two patients from Northwestern Europe in heterozygous states (Ameziane et al., 2008). A molecular study applied for Spanish patients revealed the presence of this large deletion (Castella et al., 2011). This mutation could probably be introduced in Tunisia when Andalusians began crossing the strait of Gibraltar to seek refuge in NA in 1492 but a haplotype analysis should be performed to confirm or discard this hypothesis.

Our results showed large intragenic deletions as the most frequent mutations found in homozygous and heterozygous states in 55.17% of the patients. This frequency is higher than that observed in other populations [31.25% in India (Solanki et al., 2016); 25% in Brazilian patients (Pilonetto et al., 2017)]. A meta-analysis has shown that between 15 and 40% of pathogenic mutations in *FANCA* are caused by large deletions (Ameziane et al., 2012; Gille et al., 2012). This is explained by the presence of a large number of Alu repeat sequences within *FANCA* (Levran et al., 1998; Morgan et al., 1999; Moghrabi et al., 2009). Besides the genetic structure of the *FANCA* gene, the high frequency of deletions noted among NA population is likely resulting from the presence of founder mutations.

Screening by PCR and Sanger sequencing of *FANCA* 43 exons allowed the identification of 5 different nucleotide changes in 14 FA cases (47%): one reported missense mutation (c.1304G>A; p.Arg435His), a novel missense variant (c.1258G > A; p.Asp409Glu), a nonsense mutation (c.2749C > T; p.Arg917Ter), a small deletion (c.3788-3790delTCT), and an intronic mutation (c.2222 + 166G>A). Some identified variants were described in other populations (Magdalena et al., 2005; Moghrabi et al., 2009; Solanki et al., 2016). However, none of them were described before in NA patients, except for the intronic mutation c.2222 + 166G > A (Bouchlaka et al., 2003). This mutation inherited in all FA patients originating from Kairouan (central Tunisia) represents the second most frequent mutation in our cohort (20.68%). This pathogenic variant always segregates with the same haplotype, already described (Bouchlaka et al., 2003). Our finding suggests that this intronic mutation has a common ancestor in Kairouan.

The identification of a founder mutation specific to a given geographic origin facilitates the diagnosis and reduces the diagnosis cost. In fact, various laboratories have adapted their strategies to optimize the chances of detecting the causal mutation while keeping costs to a minimum and decreasing the time required to get a reliable result.

**Figure 5:**
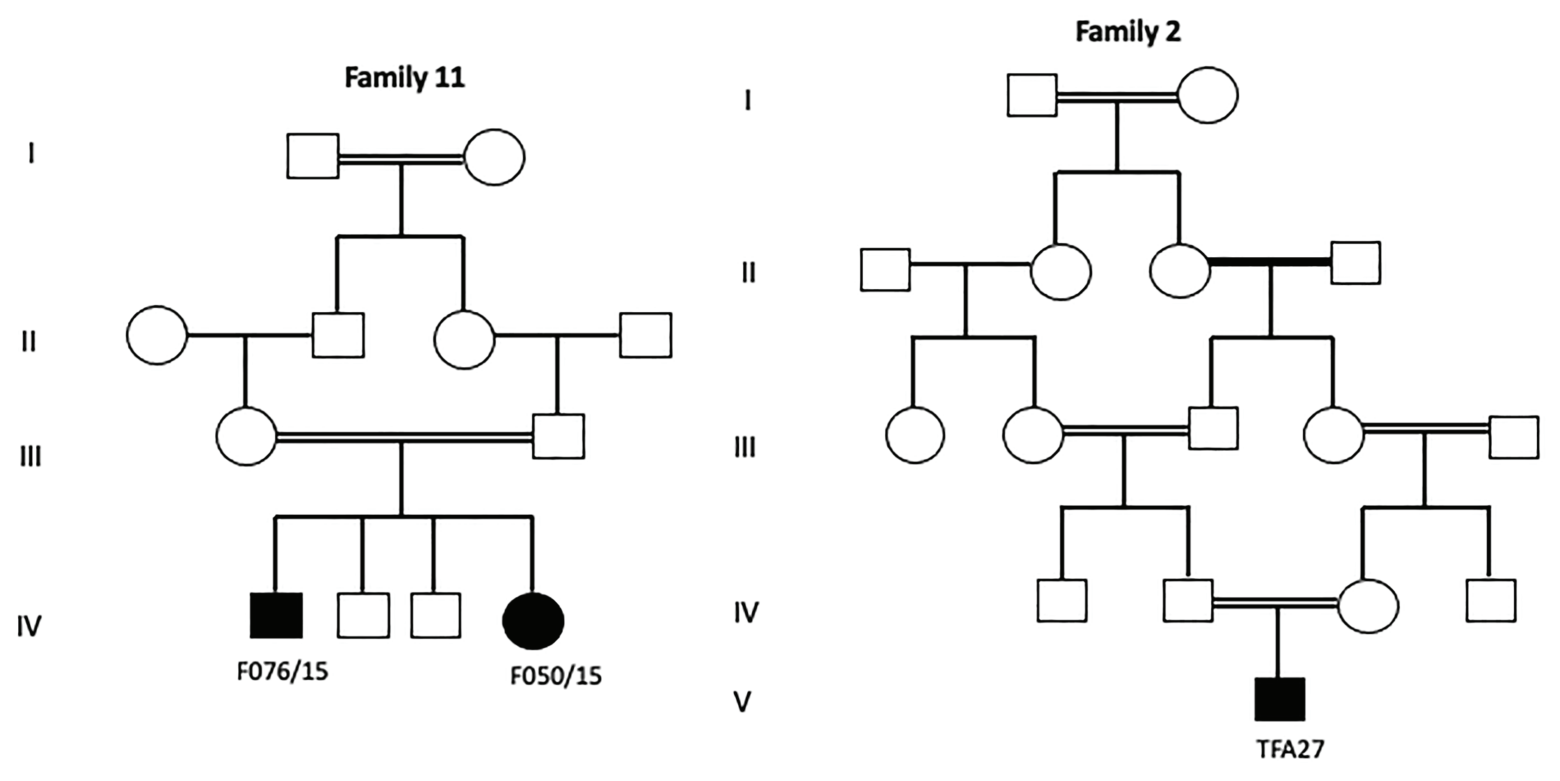
Pedigrees of two investigated FA families showing different degrees of inbreeding loops.

The identification of a founder mutation specific to a given geographic origin facilitates the diagnosis and reduces the diagnosis cost. Indeed, in Tunisia, among 174 genetic diseases with an identified molecular defect, 73 (41.9%) are due to founder mutations (Romdhane and Abdelhak, 2011; Elloumi Zghal and Chaabouni Bouhamed, 2018; Romdhane et al., 2019). This high frequency of founder mutations could be explained by the high rates of inbreeding and endogamy that characterize NA populations: 34.04% in Algeria, 37.6% in Libya and 29.9% in Tunisia (Abudejaja et al., 1987; Zaoui and Biémont, 2002; Ben Halim et al., 2013). The most frequent form of intermarriage is between first cousins, particularly paternal first cousins and includes double first-cousin marriage (Ben Halim et al., 2013). Considering the first cousins’ marriages, the risk of recessive hereditary diseases is multiplied by an average of 8 times, which doubles the total frequency of congenital and genetic diseases (Romdhane et al., 2014; Ben Halim et al., 2016). In our study, 86% of our families are consanguineous (Figure 5), which may explain the elevated number of deleterious mutations found at a homozygous state in the affected patients (82.75%). Similar values between the consanguinity rate and the percentage of homozygous mutations clearly show the impact of inbreeding on the genomic structure by increasing the number of regions of homozygosity and hence homozygous pathogenic variants.

Some of the identified *FANCA* variants (Deletion of exon 15; p.Arg917Ter; p.Arg409Glu) are shared among Tunisian, Libyan, and Algerian patients. These findings enhance the previous observations that NA patients share a common ethnic background and consequently the same mutational spectrum (Romdhane et al., 2012).

NA is characterized by its mixed population history due to migratory/invasive flows which have resulted in high levels of genetic and phenotypic variability. Our results contain private mutations, ethnic specific mutations, and recurrent founder mutations, thus reflecting a great genetic heterogeneity of *FANCA* mutations within the NA population. Indeed, prioritizing mutation screening by relying on deleterious gene variants already identified in neighboring populations can be particularly efficient. This was the case for the recurrent mutation p.V548AfsX25 affecting *XPC* and leading to *Xeroderma pigmentosum*. This mutation was firstly described in Algerian and Moroccan patients in a homozygous state (Khan et al., 2006), then another study demonstrated its high prevalence in Tunisian patients and confirmed a founder effect in this population (Ben Rekaya et al., 2009).

**Figure 6:**
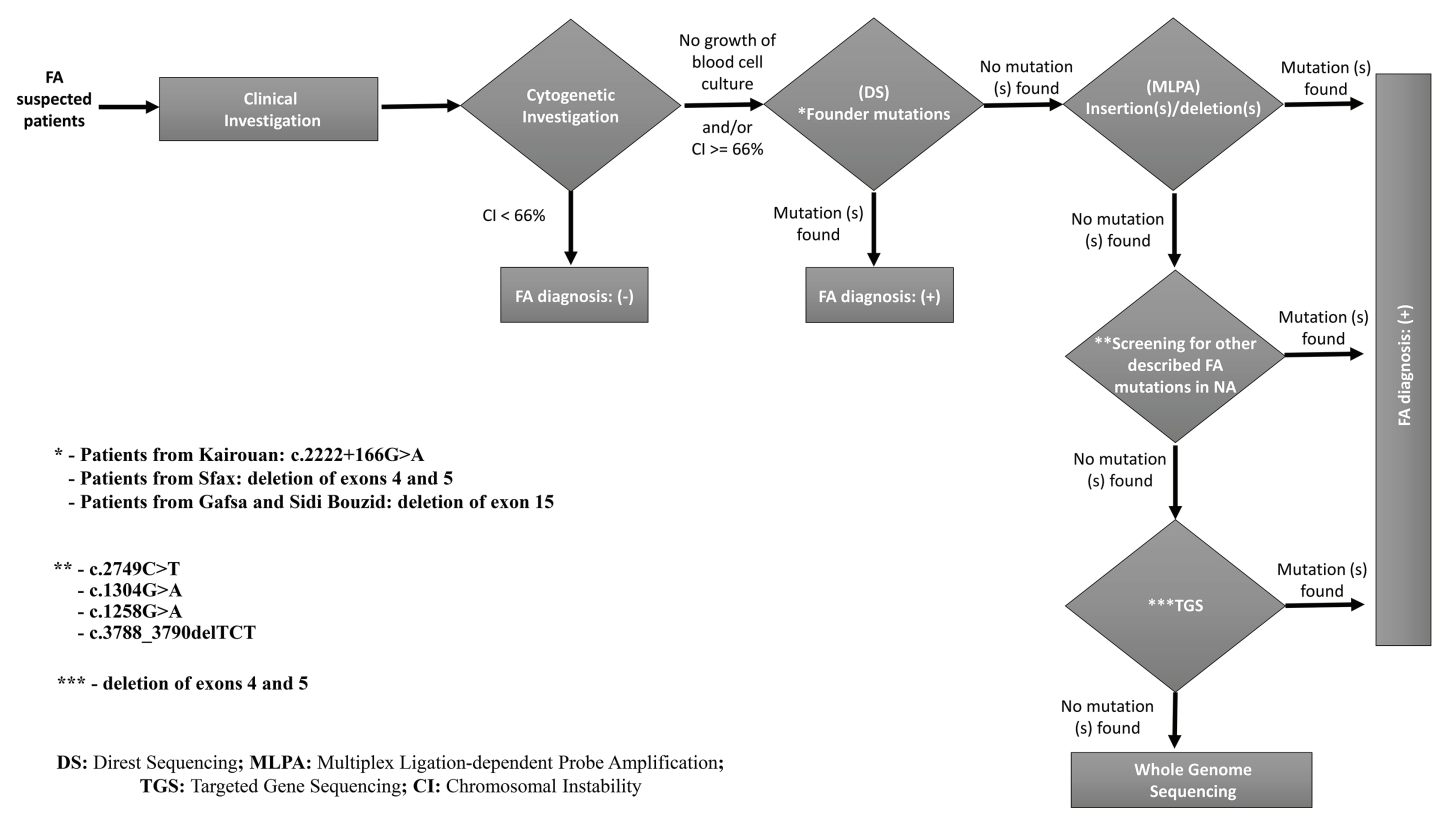
A proposed strategy for molecular diagnosis of FA in North African patients mainly from Tunisia, Algeria, and Libya.

The molecular approach applied in this study allowed us to identify causative variants for almost all patients (25 patients: 86.20%). However, the second mutated allele failed to be identified for the rest of patients (four patients: 13.79%). Taking together, we can conclude that the combination between MLPA Sanger sequencing and, possibly, TGS methods for *FANCA* mutations screening seems to be the appropriate molecular approach for a precise diagnosis of FA patients in NA (Figure 6). For patients (TFA27, GF06/09, F031/16 and F032/16) whose second mutated allele was not identified, it could be explained by (i) the presence of a second undetected pathogenic variant in intronic or regulatory regions; (ii) another FA gene could be mutated in TFA27, and the presence of *FANCA* exon 15 deletion could be due to its higher prevalence in the NA population.

The assignment of NA patients to their genetic FA subtypes and the identification of their respective mutations provide valuable data for a better knowledge of the genetic basis of FA in NA and for a more appropriate management of patients at both preventive (plan for genetic counseling and prenatal diagnosis) and therapeutic (selection of suitable donors for hematopoietic stem cell transplantation) levels.

## Data Availability Statement

Processed data related to Sanger sequencing, haplotype analysis and MLPA are available in the article. Raw data related to TGS are available from the corresponding author upon reasonable request. Indeed, in Tunisia, genetic data are considered as personal private data, for these reasons we have submitted the minimal dataset as supporting files but we are not allowed to submit the full raw data. The full raw data may be made available upon request by other investigators and after approval of our IRB.

## Ethics Statement

This work was supported by the Ethical Committee of the Institut Pasteur de Tunis (IPT) N° 2017/14/I/LR11IPT05/V0. Written informed consent for publication of both clinical data and photographs was obtained from our patients’ families. Informed consent of the legal representatives of the patient was obtained.

## Author Contributions

AA, FM, MO, and SH established the clinical diagnosis. AB and FT collected the samples. WA and AA analyzed cytogenetic results. AB and FT extracted DNA and carried out the experimental work. OM, SE, AS-G, VD, and NL analyzed molecular data. AB drafted the manuscript. AB, SA, AA, OM, MB, and JS designed the project and edited the manuscript. All authors contributed to the article and approved the submitted version.

### Conflict of Interest

The authors declare that the research was conducted in the absence of any commercial or financial relationships that could be construed as a potential conflict of interest.

## Footnotes

^1^http://primer3.ut.ee/

^2^http://genome.ucsc.edu/cgi-bin/hgPcr

^3^http://varaft.eu/index.php

^4^http://evs.gs.washington.edu/EVS/

^5^http://www.1000genomes.org/

^6^http://exac.broadinstitute.org

^7^http://www.mutationtaster.org/

^8^http://genetics.bwh.harvard.edu/pph2/

^9^http://sift.bii.a-star.edu.sg/

^10^http://folding.biofold.org/i-mutant/i-mutant2.0.html

^11^https://cadd.gs.washington.edu
